# Biogeographic history of Palearctic caudates revealed by a critical appraisal of their fossil record quality and spatio-temporal distribution

**DOI:** 10.1098/rsos.220935

**Published:** 2022-11-30

**Authors:** Loredana Macaluso, Philip D. Mannion, Susan E. Evans, Giorgio Carnevale, Sara Monti, Domenico Marchitelli, Massimo Delfino

**Affiliations:** ^1^ Dipartimento di Scienze della Terra, Università degli Studi di Torino, Via Valperga Caluso 35, 10125, Turin, Italy; ^2^ Department of Earth Sciences, University College London, WC1E 6BT London, UK; ^3^ Research Department of Cell and Developmental Biology, University College London, WC1E 6BT London, UK; ^4^ Institut Català de Paleontologia Miquel Crusafont, Universitat Autònoma de Barcelona, Edifici ICTAICP, c/ Columnes s/n, Campus de la UAB, 08193 Cerdanyola del Vallès, Barcelona, Spain

**Keywords:** amphibians, sampling biases, Urodela, Eurasia, salamanders

## Abstract

The disjunct geographical range of many lineages of caudates points to a complex evolutionary and biogeographic history that cannot be disentangled by only considering the present-day distribution of salamander biodiversity. Here, we provide a critical reappraisal of the published fossil record of caudates from the Palearctic and quantitatively evaluate the quality of the group's fossil record. Stem-Urodela and Karauridae were widespread in the Palearctic in the Middle Jurassic, suggesting an earlier, unsampled diversification for this group. Cryptobranchidae reached Europe no later than the Oligocene, but this clade was subsequently extirpated from this continent, as well as from western and central Asia. The relatively recent appearance of hynobiids in the fossil record (Early Miocene) is most likely an artefact of a taphonomic bias against the preservation of high-mountain, stream-type environments which early members likely inhabited. Salamandroids first appear in Europe, expanding into Asia by the Miocene. The apparently enigmatic and disjunct distribution of extant caudate lineages is therefore explained by a wider past geographical range, as testified by the fossil record, which was fragmented during the late Cenozoic by a combination of tectonic (i.e. the uplift of the Tibetan Plateau) and climatic drivers, resulting in regional extirpations.

## 1. Introduction

Caudata, the total-group that comprises extant salamanders (Urodela) and stem-representatives, is a rich and diverse clade of lissamphibians, currently numbering *ca* 770 living species [1,2]. Nearly all of their present-day diversity is concentrated in the Holarctic, with a small number of species endemic to South America and southeast Asia [3]. This distribution largely reflects the evolutionary history of a group that first appeared and diversified in the northern part (Laurasia) of the supercontinent Pangea at the height of its Mesozoic fragmentation; thus, the group seemingly had little time to colonize its southern counterpart, Gondwana, prior to its separation [1]. Despite the large geographical area represented by the Palearctic ecozone (comprising Eurasia, north of the Himalayas, as well as northern Afro-Arabia) within the Holarctic realm, only a small portion of the taxonomic diversity of extant caudates currently inhabits it, representing approximately 15% of species (98 of 770) and 37% of genera (24 of 65), and including members of five families [4]. Instead, much of the extant diversity of Caudata is present in the Nearctic ecozone, which covers most of North America, and mostly derives from the radiation of the family Plethodontidae. Interestingly, some families are present in both the Nearctic and Palearctic ecozone. Cryptobranchidae and Hynobiidae are sister taxa and represent the two extant families of the Cryptobranchoidea [5]. Whereas cryptobranchids are present in both North America and Asia, hynobiids are restricted to Eurasia [3]. All other extant urodelans are part of Salamandroidea, the most speciose clade, including several families endemic to the Americas [6]. Many salamandroid lineages show a disjunct geographical range spanning North America and Eurasia (e.g. Plethodontidae, Proteidae and Salamandridae).

These distributional patterns point to a complex evolutionary and biogeographic history that cannot be disentangled by considering only the present-day distribution of caudate biodiversity, which represents only a temporal snapshot that has been overprinted by extirpations and anthropogenic disturbances. Information about the caudate fossil record is scattered across an extensive literature, contrasting with syntheses documenting extant species (see e.g. [3,7] and the literature therein). Crucially, there have been no recent attempts to extensively evaluate the evolutionary history of the group through time and space, with the last systematic review of its global fossil record produced by Estes [8], over 40 years ago, and partially updated by Milner [9], who included part of Eurasia, northern Africa and the Middle East. The European fossil record was reviewed and evaluated by Roček [10], and Gao *et al*. [11] worked on synthesizing the Jurassic and Cretaceous salamander occurrences in China. Several subsequent studies have focused on the evolution and biogeography of caudates from smaller geographical areas, time intervals and/or sub-clades (e.g. [12–14]). The absence of an up-to-date, taxonomically and spatio-temporally more inclusive dataset limits our ability to synthesize this information, precluding an evaluation of the macroevolutionary and biogeographical history of caudates. As a first contribution to rectify this, here we provide a critical reappraisal of the published fossil record of caudates from the Palearctic, including occurrences of extant species. In tandem, we quantitatively evaluate the quality of the group's fossil record, to better understand how the distribution of caudate diversity has been influenced by sampling biases through time and space.

## 2. Material and methods

Data on the distribution of Palearctic caudates were extracted from the literature, including resources such as *The Paleobiology Database* (https://paleobiodb.org), as a starting point. We included occurrences of fossil remains that have been formally described and/or figured. For each caudate-bearing locality, the skeletal completeness of each distinct taxon was quantified based on the presence of elements of the different skeletal regions (i.e. skull, lower jaw, hyoid, vertebrae, ribs, limbs and girdles), with a score of ‘one' if at least one element of the relative region is preserved (electronic supplementary material, appendix S1). Subsequently, these values were summed for each region, and a mean average completeness value was calculated for each time bin, normalized to 100 (see electronic supplementary material, appendix S2). To account for heterogeneous sampling of different time bins, we also calculated the ratio of the number of occurrences per time bin relative to the number of caudate-bearing localities. Taxonomic re-identifications based on our work not commented upon in the main text are reported in electronic supplementary material, appendix S3. As the taxonomic identification of fossil remains is, in the first instance, guided by our knowledge of diagnostic characters and variation among extant species, it is likely influenced by how extensively the different regions of skeletons of extant species have been studied. To detect possible publication biases concerning the osteology of extant species, we evaluated the information on the different skeletal regions of extant urodele species present in the literature (following the approach of [15]), comparing these data with the skeletal regions described from fossil localities. The subdivision of the cranium into skull and lower jaw follows Hildebrand [16]. The complete reference list used to estimate the current knowledge of the osteology of extant species is provided in electronic supplementary material, appendix S4. Distribution maps were produced with QGIS 3.12.1 [17].

## 3. Results

### 3.1. Distribution of spatio-temporal and osteological data

**Figure 1.**
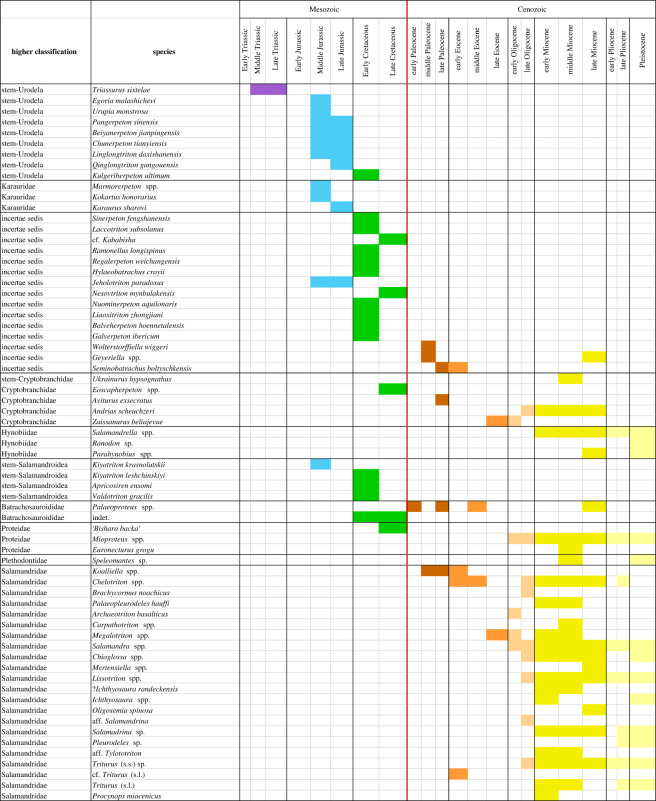
Stratigraphic ranges of fossil occurrences of Palearctic taxa of caudates. **spp.** = stratigraphic range referred to more than one species belonging to the genus; **sp.** = record with identification at the genus level. See electronic supplementary material, appendix S1 for details about localities.

**Figure 2.**
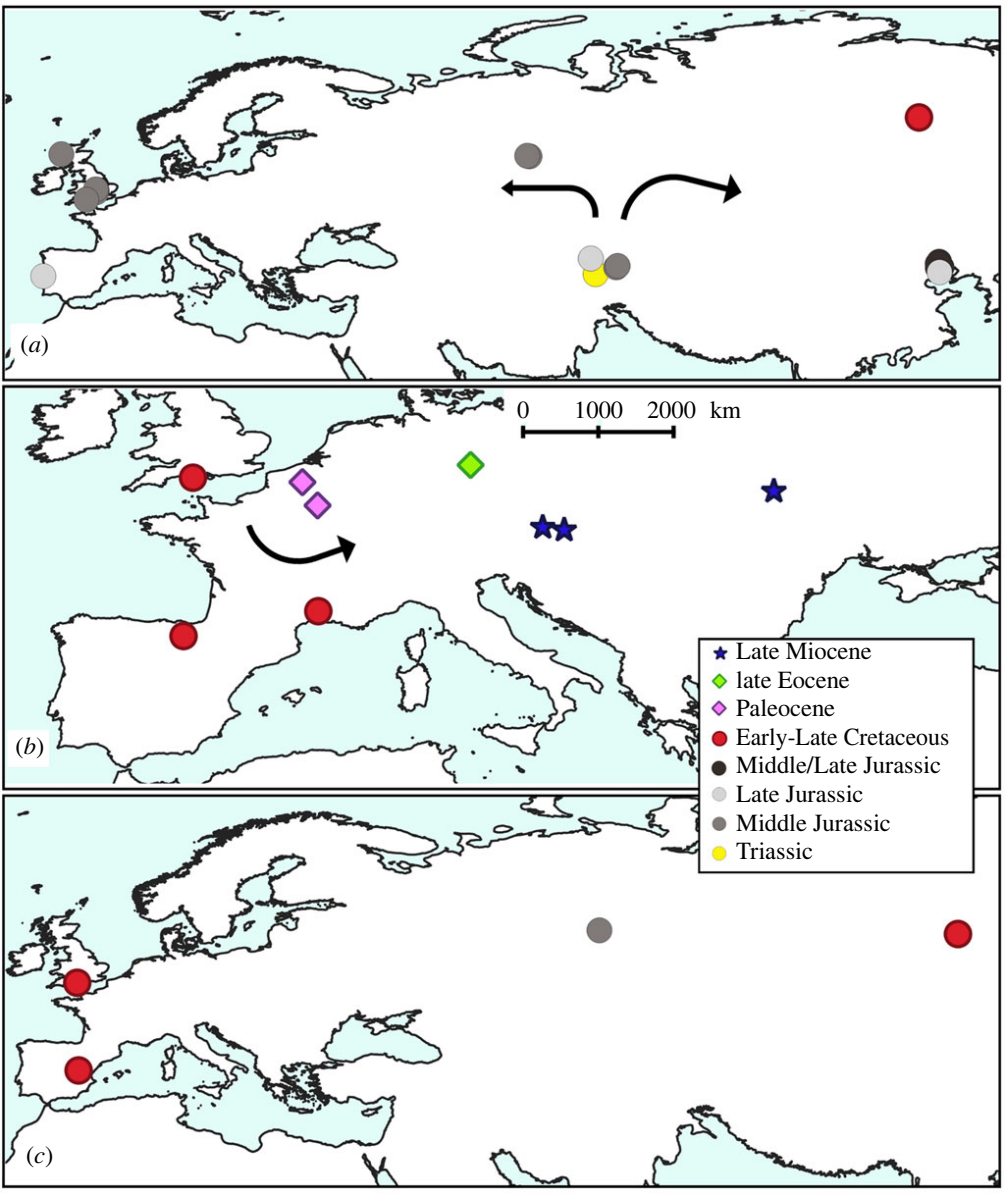
Map of fossil localities yielding remains of Palearctic caudates: (*a*) stem-Urodela and Karauridae, (*b*) Batrachosauroididae and (*c*) possible stem-Salamandroidea. See electronic supplementary material, appendix S1 for details about taxa and localities. Black arrows indicate the possible dispersal routes of the group suggested by the stratigraphic range of the fossil occurrences. Localities with stratigraphic ranges with uncertainty or laying on the boundary between two periods were labelled as pertaining to the older period (e.g. ‘Middle–Late Jurassic’ was labelled as ‘Middle Jurassic’, but see electronic supplementary material, appendix S1 for the exact range of each locality).

**Figure 3.**
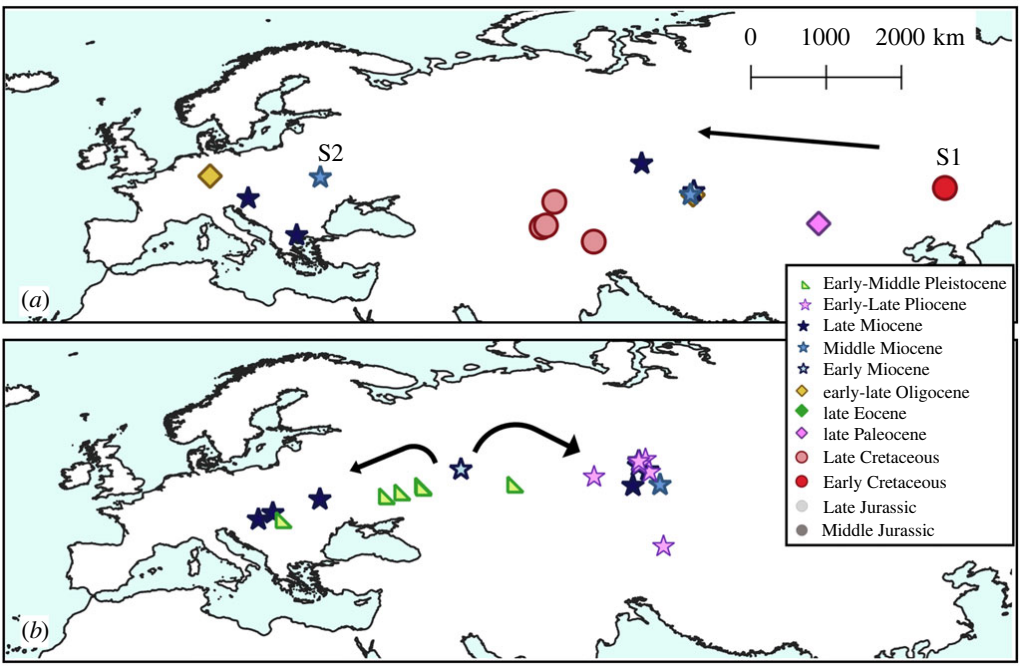
Map of fossil localities yielding remains of Palearctic Cryptobranchoidea: (*a*) Cryptobranchidae and (*b*) Hynobiidae. S1 = stem-Cryptobranchoidea (*Nuominerpeton aquilonaris*); S2 = stem-Cryptobranchidae (*Ukrainurus hypsognathus*). See electronic supplementary material, appendix S1 for details about taxa and localities. Black arrows indicate the possible dispersal routes of the group suggested by the stratigraphic range of the fossil occurrences. Localities with stratigraphic ranges with uncertainty or laying on the boundary between two periods were labelled as pertaining to the older period (e.g. ‘Middle–Late Jurassic’ was labelled as ‘Middle Jurassic’, but see electronic supplementary material, appendix S1 for the exact range of each locality).

**Figure 4.**
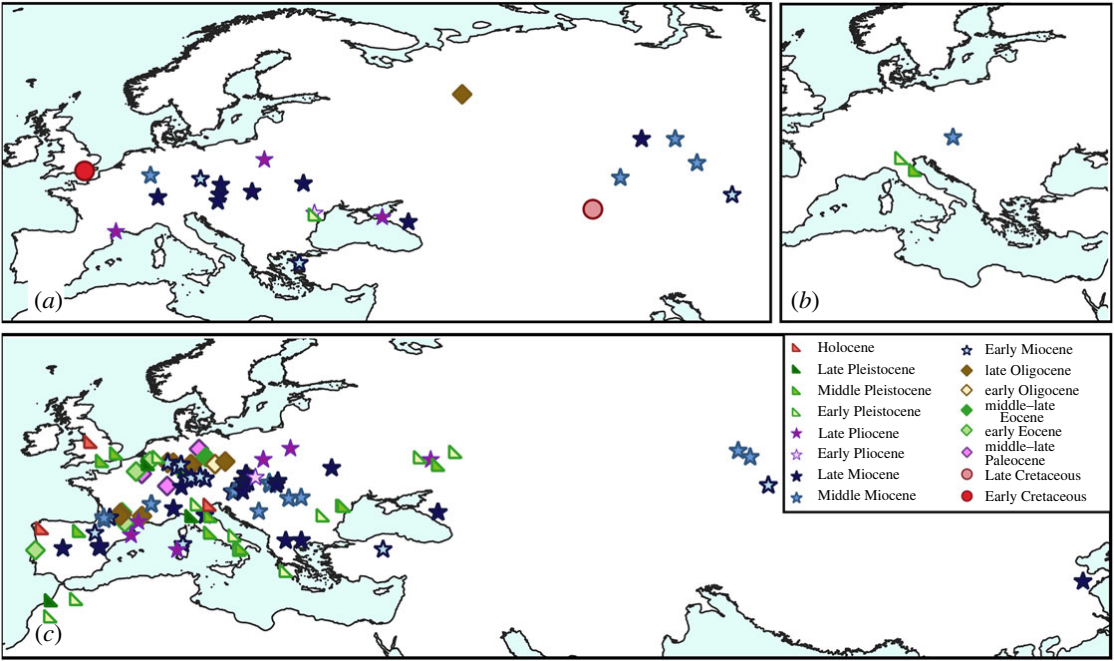
Map of fossil localities yielding remains of Palearctic Salamandroidea: (*a*) Proteidae, (*b*) Plethodontidae and (*c*) Salamandridae. See electronic supplementary material, appendix S1 for details about taxa and localities. Black arrows indicate the possible dispersal routes of the group suggested by the stratigraphic range of the fossil occurrences. Localities with stratigraphic ranges with uncertainty or laying on the boundary between two periods were labelled as pertaining to the older period (e.g. ‘Middle–Late Jurassic’ was labelled as ‘Middle Jurassic’, but see electronic supplementary material, appendix S1 for the exact range of each locality).

**Figure 5.**
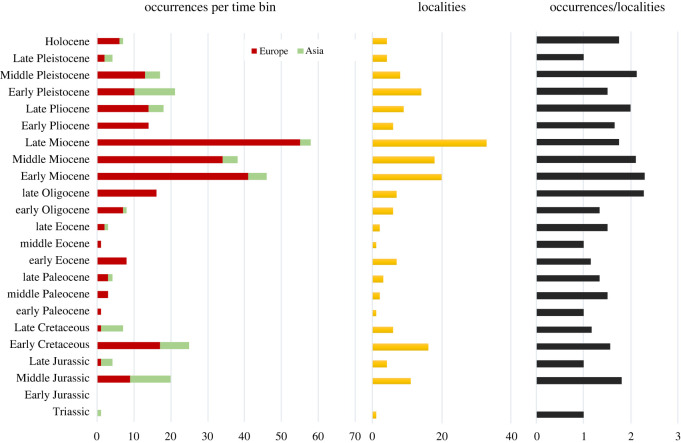
Total occurrences per time bin from each continent. Number of localities per time bin in yellow. Average diversity per locality per time bin (ratio of occurrences/localities). Localities with ranges of uncertainty or with ages on the boundary between two periods were counted in the older period (e.g. ‘Middle*–*Late Jurassic' was counted in the column of ‘Middle Jurassic'). See electronic supplementary material, appendix S1 for details about taxa and localities.

**Figure 6.**
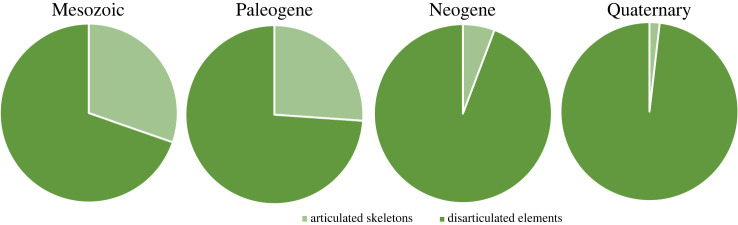
Fossil occurrences preserved as articulated skeletons versus disarticulated skeletal elements. See electronic supplementary material, appendix S1 and S2 for details about taxa and localities.

**Figure 7.**
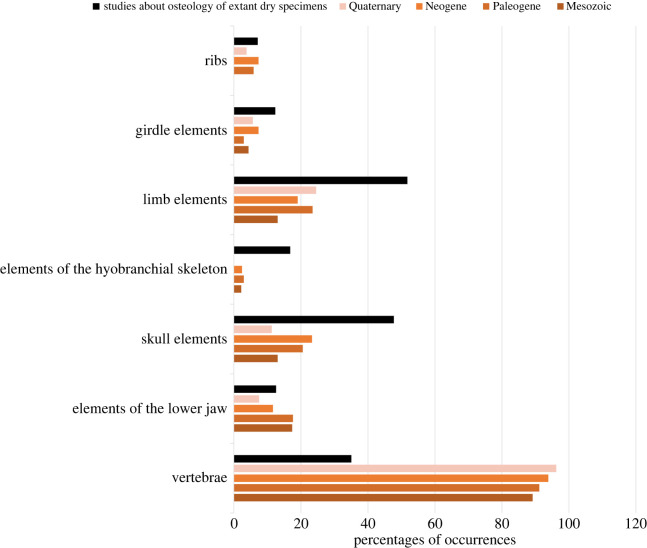
Percentages of occurrences preserved as disarticulated elements of the different skeletal regions (vertebrae, lower jaw, skull, hyobranchial skeleton, limbs, girdles and ribs). Percentages are calculated on the basis of the total occurrences of remains preserved as disarticulated elements, which is to say the total occurrences minus the occurrences preserved as articulated skeletons. See electronic supplementary material, appendix S2 for more details. Black columns refer to the osteological information present in the literature, listed in electronic supplementary material, appendix S4, concerning extant species of Palearctic urodeles (see [15] for further details).

A total of 234 taxonomic occurrences (taxon per locality data) from European localities and 86 from Asian localities are recognized, and only six occurrences from northern Africa. The stratigraphic range for each species is presented in figure 1 and the spatio-temporal distribution of each taxonomic group is presented in figures 2–4. Most of the occurrences are from the Neogene (167 occurrences), 55 occurrences known from the Quaternary, 42 from the Paleogene, 36 occurrences from the Cretaceous, 25 from the Jurassic and just one from the Triassic (figure 5). The vast majority of fossil remains (*ca* 87% of the occurrences) is represented by disarticulated elements and not by complete or partially articulated skeletons (figure 6). Among these, vertebrae are almost always present (present in *ca* 93% of the occurrences represented by disarticulated elements), followed by skull elements and limbs (present in *ca* 20.7% and 21% of occurrences, respectively), and this is constistent in all time bins (figure 7). In the published literature, most of the information about extant urodelan osteology is focused on limbs and skulls (*ca* 50% of the references include data on these districts), whereas vertebrae of extant Palearctic salamanders are less frequently described (in *ca* 35% of the references) (figure 7).

### 3.2. Palearctic fossil record

#### 3.2.1. Triassic–Jurassic

The stratigraphically earliest known species of caudate from Eurasia (as well as of the planet) is *Triassurus sixtelae*, from the Middle/Late Triassic of Kyrgyzstan [18,19]. No further caudate specimens have been identified from the Triassic of Eurasia, and the group also lacks an Early Jurassic record from this region. The only possible report from the Early Jurassic consists of three mandibles from India reported as being from sirenids [20], but they do not belong to an urodele according to Milner [21] and Gardner [22]. A rich caudate record is documented from Middle Jurassic strata, with most taxa described from Asia [13,23] (figure 2). This comprises *Kokartus honorarius* from Kyrgyzstan [24,25], the stem-urodeles *Urupia monstrosa* and *Egoria malashichevi* from Russia [26,27], as well as *Kiyatriton krasnolutskii*, a possible cryptobranchoid from Russia ([28], but see the ‘Early Cretaceous' section below for a different interpretation) (figure 1). As noted by Skutschas [13], the Russian species *U. monstrosa* shows close affinities to the genus *Marmorerpeton*, from the Middle Jurassic of the UK, in particular the species *M. freemani* [29], whereas *E. malashichevi* (from the same assemblage as *U. monstrosa*) is strikingly similar to a second species of *Marmorerpeton*, *M*. *kermacki* [29]. These similarities suggest that these Russian and UK species could belong to the same lineage of stem-salamanders, which would indicate that it was already widely dispersed across Eurasia by the Middle Jurassic. Recent analyses confirm the attribution of *Marmorerpeton* and *Kokartus*, at least, to the Karauridae ([30]; see below). A fragmentary trunk vertebra from the Middle Jurassic of Russia has been tentatively identified as an indeterminate salamandroid [31], but more complete material will be required to confidently determine its affinities. Currently, *Marmorerpeton* is the only named caudate genus from the Middle Jurassic of Europe, although contemporaneous specimens belonging to two additional taxa (informally known as ‘Kirtlington Salamander A' and ‘Kirtlington Salamander B') have also been documented from the UK [32–34] and are currently under study. Fossils of caudates are rare on the African continent and they remain mostly undescribed. Reported occurrences from the Triassic–Jurassic interval are limited to the Middle Jurassic of Morocco and Madagascar, but their fragmentary nature means that it is uncertain whether they are even attributable to Caudata [35,36].

Several genera and species are known from the Middle/Late Jurassic boundary of China, including the stem-urodeles *Chunerpeton tianyiensis* [30,37,38] and *Neimengtriton daohugouensis* [30,39,40]. *Beiyanerpeton jianpingensis* [41] and *Qinglongtriton gangouensis* [42] were recovered in an early-branching position within Salamandroidea by Jia & Gao [43], whereas *Linglongtriton daxishanensis* [43] and *Pangerpeton sinensis* [44] were placed on the stem-hynobiid lineage [43]. However, all these taxa were identified as stem-urodeles in the recent analysis of Jones *et al*. [30]. In general, Jurassic taxa from China are mostly known from slabs, often as skeletal impressions, making it difficult to evaluate their relationships with other fossil species that are only known from isolated, three-dimensional remains (but see [44] and [43] for detailed studies of their possible phylogenetic relationships). No Late Jurassic caudate remains have currently been described from Europe or Afro-Arabia. *Karaurus sharovi* was described from the Late Jurassic of Kazakhstan by Ivakhnenko [18], who placed it in a new family, Karauridae. In addition to some indeterminate remains from the Middle Jurassic of Russia [45,46], both *Kokartus* and *Marmorerpeton* can be attributed to this family (see above).

#### 3.2.2. Early Cretaceous

The Early Cretaceous European fossil record is far richer than its Jurassic counterpart (figure 2), with *Apricosiren ensomi* from the UK [47], *Balveherpeton hoennetalensis* from Germany [48], *Galverpeton ibericum* and *Valdotriton gracilis* from Spain [49,50] and *Hylaeobatrachus croyi* from Belgium [8,51]*. Apricosiren* and *Valdotriton* have been suggested to be closely related taxa, placed as early diverging members of Salamandroidea [47]. One recent phylogenetic analysis, however, recovered *Valdotriton* as sister taxon of the extant North American ambystomatid salamandroid genus *Ambystoma* [43], whereas the analysis of Jones *et al*. [30] placed *Valdotriton* on the salamandroid stem. The morphology of the vertebrae (including the atlas) of *Valdotriton* and *K. leshchinskiyi* is similar, whereas the morphology of the other known species of *Kiyatriton*, *K. krasnolutskii* (see above) shares features with the morphologically plesiomorphic family Ambystomatidae, notably the unseparated articular surface of the odontoid process, the flat occipital cotyle, as well as the length (relative to the width) of the centrum (see [52]: figure 2). Based on these considerations, we agree with Jones *et al*. [30] that *Valdotriton* belongs to the stem of Salamandroidea and we suggest that *Kiyatriton* and *Apricosiren* might also occupy a similar phylogenetic position.

Both *Hylaeobatrachus*, known from a single larval or paedomorphic specimen, and *Galverpeton*, known from a single trunk vertebra, have an uncertain placement within Caudata [49,50]. In the case of this latter taxon, it appears to be most similar to extant early branching North American plethodontids, given the presence of opisthocoely and a single neural crest with a posterodorsal bulge; however, the trunk vertebra lacks a fully enclosed spinal nerve, which is a synapomorphy of Plethodontidae [53], and its placement is therefore uncertain.

Several approximately contemporaneous caudate occurrences from the UK have been identified as distinct taxa, but they have yet to receive a formal name and have generally been referred to as ‘Ashdown type 1–4 salamanders', ‘Keymer salamander' and ‘Wessex Formation type 1–3 salamanders', referring to the horizons that yielded them [54]. The Ashdown type 2, 4 and Keymer salamanders share some features with *Balveherpethon*, including a thick and strongly elongated odontoid process and a dorsoventally compressed, shallowly concave anterior cotyle, but also show differences. The morphology of these atlantes closely resembles that of the scapherpetontid species *Lisserpeton bairdi* from the latest Cretaceous of North America [55]. Post-atlantal vertebrae referred to *Balveherpeton* also show similarities with this extinct family (and *Lisserpeton* in particular), including the presence of a ventral keel and anterior and/or posterior basapophyses [48]. Previous authors have mentioned the presence of Scapherpetontidae in the Mesozoic and Cenozoic of Europe but have not described or figured this material [56,57]. *Balveherpeton* and at least some of the UK remains could be related to Scapherpetontidae, but the UK atlantes show a pronounced elongation that differentiate them from those of scapherpetontids (characterized by short atlantes) making them somewhat more similar to Ambystomatidae and stem-salamandroids. These taxa are therefore retained as *incertae sedis* within caudates pending further study, especially with regards to variability in extant ambystomatids and extinct scapherpetontids. Remains referred to an indeterminate taxon of the otherwise primarily North American extinct family Batrachosauroididae were reported from the Early Cretaceous of the UK [47].

Among the UK remains described by Sweetman & Evans [54] is a postatlantal vertebra, BEXHM: 2010 16.14, with an unusual morphology. This specimen shares characters with some extant proteid species, including a strongly posteriorly projecting neural arch that extends beyond the postzygapophyses, and the combination of a hollow parapophysis and solid diapophysis [58]. Whereas it differs from the European lineage of *Mioproteus*/*Proteus* (see below), BEXHM: 2010 16.14 shares these (and other) features with the extant North American genus *Necturus*, whose fossil record extends back to the late Paleocene of Canada [58]. Because of this close similarity, it is possible that BEXHM: 2010 16.14 represents an early occurrence of a proteid lineage distinct from *Mioproteus*/*Proteus*, possibly leading to the species *Euronecturus grogu*, recently described from the Middle Miocene of Germany ([59]; see below).

*Ramonellus longispinus*, from the Early Cretaceous of Israel [60], represents a possible caudate of uncertain affinities and is the sole pre-Pleistocene fossil occurrence of the group known from the Arabian Peninsula or Levant region [36]. No caudate remains have been described from the Early Cretaceous of Africa [36].

The Asian record is rich in the Early Cretaceous. Chinese taxa are represented by the proposed stem-hynobiids *Nuominerpeton aquilonaris* and *Liaoxitriton zhongjiani* [42,61], the possible salamandroid *Regalerpeton weichangensis* [62,63], as well as *Jeholotriton paradoxus* [64] (figures 1 and 3). However, the phylogenetic positions of all of these Chinese species are uncertain [43], with cryptobranchoid [65], salamandroid [63] and stem caudate [30] affinities proposed. *Sinerpeton fengshanensis* and *Laccotriton subsolanus* are non-salamandroid caudates of uncertain placement described by Gao & Shubin [66] and Gao *et al*. [67], respectively. A stratigraphically younger species of the putative salamandroid *Kiyatriton* (*K*. *leshchinskiyi*) is recognized from the Early Cretaceous of Russia [68,69], as is the stem-urodele *Kulgeriherpeton ultimum* [70].

#### 3.2.3. Late Cretaceous

The European record of Late Cretaceous caudates is scarce, limited to indeterminate and/or undescribed remains from France, Portugal and Spain (e.g. [71–74]). Among these, Garcia *et al*. [73] proposed that a specimen from France has possible plethodontid affinities, but a Batrachosauroididae affinity cannot be ruled out based on the published descriptions. Generically indeterminate remains of this family have also been described from a coeval locality in France [75], and indeterminate remains described by Duffaud & Rage [72] from Spain might also be referrable to this family.

There is a slightly richer record from Asia, although these occurrences are restricted to Central Asia [13,23]. Several localities have yielded remains attributed to the cryptobranchid *Eoscapherpeton*, with *E*. *asiaticum* and *E*. (*Horezmia*) *gracilis* recognized in Uzbekistan [13,76], and specifically indeterminate remains from Kazakhstan [23] and Tajikistan ([13]; originally described as *E*. *superum* by Nessov [77]). Cryptobranchoid affinities have also been suggested with caution for *Nessovtriton mynbulakensis*, from Uzbekistan [13,78]. Finally, Nessov [24,77] described *Bishara backa* from Kazakhstan, which he tentatively identified as a prosirenid. Skutschas [13] noted that the known material of *Bishara backa* (a partial atlas, now lost) could be referred to the family Proteidae. An additional atlas figured by Skutschas [13] bears a striking similarity with the extinct proteid *Mioproteus* [79,80], whereas it clearly differs from both *Euronecturus* and *Necturus* [59]. As such, we agree with Skutschas [13] that *Bishara backa* potentially represents another proteid lineage in the Cretaceous of Eurasia.

Several occurrences of caudate have been documented from the Late Cretaceous of Africa [36]. Two species of *Kababisha* (*K*. *humarensis* and *K*. *sudanensis*) were described by Evans *et al*. [81] from the Late Cretaceous of Sudan. As Sudan is not part of the Palearctic ecozone, these species were not included in the dataset used for the completeness analysis (electronic supplementary material, appendix S1), but they are discussed here due to their uniqueness, as the only caudates (extinct or extant) ever reported south of the Sahara Desert. Morphologically, *Kababisha* shows a close affinity with the extant North American early-branching salamandroid family Sirenidae [81]. Fossil material from the Late Cretaceous of Niger and Morocco has been regarded as similar to this genus (cf. *Kababisha*; [82,83]). No stratigraphically younger caudate fossil occurrences have been described from Africa prior to the Pleistocene.

#### 3.2.4. Paleocene

The Paleocene record of Eurasian caudates is scarce. The stratigraphically oldest remains come from the early Paleocene Hainin site in Belgium [84–86], with material attributed to *Palaeoproteus gallicus* [87], a member of the enigmatic batrachosauroidid clade (figures 1 and 2). *Palaeoproteus gallicus* was firstly described from the late Paleocene of northeastern France [87,88] (figure 2). The highest diversity of Eurasian caudates from this epoch is represented at the middle Paleocene Walbeck locality in northeastern Germany, with three puzzling caudate taxa (*Geyeriella mertensi*, *Koalliella genzeli* and *Wolterstorffiella wiggeri*) described by Herre [89].

*Geyeriella mertensi* is represented in this fossil assemblage by 19 disarticulated post-atlantal vertebrae and was referred to the salamandroid ambystomatid subfamily Dicamptodontinae (genus *Dicamptodon*, currently belonging to the Dicamptodontidae family) by Estes [8]. These vertebrae share several characters with the Mesozoic taxon *Valdotriton*, including double-headed transverse processes, anterior basapophyses and a posteriorly projecting neural arch that results in a short, horizontal neural spine. Estes [8] suggested that *Geyeriella* might be congeneric with the salamandroid *Bargmannia wettsteini*, from the Late Miocene of Slovakia [90], and here we agree with his interpretation. From the same Paleocene locality as *G. mertensi*, an atlas was referred by Herre [89] to the salamandrid *Koalliella genzeli*, but, although we agree with him that the morphology of both trunk and caudal vertebrae attributed to *Koalliella* shows the typical salamandrid morphology, the atlas does not exhibit diagnostic characters that would support this attribution. The unseparated articular surface of the odontoid process, as well as the flat, vertical occipital cotyle, means that the atlas is most similar to those of *Kiyatriton krasnolutskii* and *Geyeriella wettsteini* [28,90]. Therefore, we suggest that the atlas referred by Herre [89] to *Koalliella genzeli* is more likely to belong to *Geyeriella mertensi*. These atlantes have the same plesiomorphic salamandroid atlantal morphology as those of *Valdotriton* and *Kiyatriton*, and resemble that of the extant genus *Ambystoma* [52], pointing to a possible stem-salamandroid position for these remains (figure 2*c*).

*Wolterstorffiella wiggeri* appears to have a close affinity with Cryptobranchoidea, based on a suite of characters, including amphicoelous vertebrae, absence of ventral keel and basapophyses, confluent diapophyses and parapophyses, and a posterodorsal bulge formed by the neural arch and the horizontal neural spine in trunk vertebrae, which is replaced in the caudal vertebrae by two divergent bulges ([89]: figures 1, 4, 5). The overall size of the vertebrae figured by Herre [89] points to a cryptobranchid affinity; however, the relatively small vertebral centrum (compared with the neural arch) is closer to that of hynobiids, including the extant taxon *Salamandrella* [91], as well as the extinct genus *Parahynobius* (see below; [92]). *Wolterstorffiella* is therefore herein considered a putative cryptobranchoid species, even though only first-hand observation of the material can confirm this attribution (i.e. the absence of the foramina for the exit of the spinal nerve needs to be confirmed).

*Koalliella genzeli* is known solely from isolated vertebrae, showing the typical morphology of salamandrids ([89]: fig. 11). In particular, these vertebrae are characterized by the dorsally enlarged neural crest typical of the extinct Neogene Eurasian taxa *Chelotriton* and *Lissotriton rohrsi* (see below), as well as extant crocodile-newts from southeast Asia (i.e. the *Tylototriton*/*Echinotriton* group; [93,94]).

Two isolated vertebrae from Cernay (late Paleocene of France) were identified as *Koalliella* sp. by Estes *et al*. [88]. One of these vertebrae differs from the *Koalliella* type material (as well as the other Cernay vertebra) in that it has posterior basapophyses. However, this is perhaps not surprising, given that several salamandrid species are characterized by posterior basapophyses in the first or second caudosacral vertebrae, representing the first bulge of the haemal arch [95]. This interpretation of vertebral position is supported by the transverse processes in the Cernay specimen, which are dorsoventrally developed (forming a vertical lamina in lateral view) and lack articulations for ribs, as is typical for non-rib-bearing caudosacral vertebrae. The general outline of the vertebra figured by Estes *et al*. [88] is indeed similar to *Koalliella genzeli*, but also shows some similarities with the North American salamandrid genus *Taricha*, including the extinct species *T*. *miocenica* and *T*. *oligocenica* [8]. In particular, it shares the presence of lateral eversion of the dorsal capping plates (*sensu* [96]) covering the neural crest, which also characterizes the extant European species of *Salamandrina*. The attribution to *Koalliella* is herein tentatively supported, pending further study on its relationship with other genera of caudates that show vertebral capping on the neural crest.

A putative salamandrid (cf. *Salamandra* sp.) was also reported from the Cernay assemblage by Estes *et al*. [88], further supporting the presence of that clade in the Paleocene of Europe. However, it is highly unlikely that this specimen is referrable to *Salamandra*, as the ventral lamina of the vertebra is expanded, contrasting with the absent or reduced ventral crest that typically characterizes vertebrae of *Salamandra* [95]. The material is too fragmentary to provide a generic attribution and is herein regarded as an indeterminate specimen belonging to Salamandridae.

*Seminobatrachus boltyschkensis* is known from multiple skeletons from Ukraine; however, its stratigraphic age is poorly constrained, and it could be late Paleocene or early Eocene [65] (figure 1). The affinities of *Seminobatrachus* are uncertain, but the phylogenetic analysis of Skutschas & Gubin [65] suggested a close relationship with Ambystomatidae. An unequivocal attribution to this family was excluded by the presence of a ventral keel on trunk vertebrae, but this structure is highly variable and evolved and disappeared several times independently in different salamander groups (e.g. Batrachosauroididae, Proteidae). Furthermore, the vertebrae of the putative stem-salamandroid *Geyeriella* are also characterized by the presence of a ventral keel [89,90].

The Asian record is limited to a single locality in the late Paleocene of Mongolia (figure 3*a*), which has yielded the cryptobranchid *Aviturus exsecratus* [97,98]. A second species, *Ulanurus fractus*, was also described from this locality by Gubin [97], but this has since been synonymized with *Aviturus exsecratus* by Vasilyan *et al*. [99].

#### 3.2.5. Eocene

A second species of the batrachosauroidid, *Palaeoproteus*, was described from the middle Eocene of Germany by Herre [100]. This species, *P*. *klatti*, is known from almost complete skeletons [100].

The salamandrid *Chelotriton* is one of the most common taxa in European vertebrate assemblages, spanning the middle Eocene through to the Pliocene (figures 1 and 4*c*; [8,14,79,101–120]). According to Marjanović & Witzmann [115], *Chelotriton* is an early-branching member of the extant salamandrid group Pleurodelinae (the sub-family including all extant ‘newts'). The taxonomic history of this genus is complex and controversial. Most of the remains attributed to this genus have been referred to the species *Chelotriton paradoxus*, originally described by Pomel [121] from the late Oligocene and Early Miocene of France and Germany, respectively. Estes [8] synonymized several taxa from the Oligocene–Miocene of Germany with *Chelotriton paradoxus*, namely *Tylototriton primigenius* [122], *Heliarchon* [123], and *Grippiella*, *Palaeosalamandrina* and *Tischleriella* (all described by Herre [124]). The species *Chelotriton robustus* was described based on a single, complete and articulated skeleton from the middle Eocene of Germany by Westphal [125]. Nussbaum & Brodie [126] considered the species *Tylototriton weigelti* [100], from the middle Eocene of Germany, as a valid species of the extant pleurodeline Asian genus *Tylototriton*; however, Roček [127] argued that this species should be referred to *Chelotriton*, with which we agree. Whether these Eocene remains should be assigned to *C. paradoxus* or they belong to one or two separate species requires further study. Pending detailed descriptions of the specimens, we tentatively consider *C*. *robustus* and *C*. *weigelti* as valid species of *Chelotriton*. *Chelotriton ogygius* (= *Salamandra ogygia sensu* [128] = *Polysemia ogygia sensu* [123] = *Epipolysemia ogygia sensu* [129]) was described based on a nearly complete skeleton from the Early Miocene of Germany [8] and has been regarded as a nomen dubium by many authors (e.g. [116]). Given that the type material seems to be lost, Estes [8] argued that possible synonymy of *C. ogygius* with *C. paradoxus* cannot be conclusively demonstrated. As such, we tentatively retain *C. ogygius* as a distinct species of *Chelotriton*.

An indeterminate salamandrid was described from the early Eocene of Portugal by Rage & Augé [130]. The vertebrae of this specimen show the typical cap of the neural crest of *Koalliella* and *Salamandrina*, and a general morphology compatible with these two taxa. Based on the known stratigraphic distributions of these two genera, these remains are herein referred to cf. *Koalliella* sp., pending further study clarifying the relationship of this taxon. A vertebra from the early Eocene of Dormaal, Belgium, was described as similar to *Tylototriton* by Hecht & Hoffstetter [131]. However, it was assigned to *Koalliella* sp. by Godinot *et al*. [132] and Estes [8]. From the same time interval, Augé *et al*. [133] described vertebrae with a similar dermal capping of the neural crest from France, referring them to an indeterminate salamandrid. As these specimens were not figured, we refrain from providing a more refined attribution than Salamandridae indet, but it is possible that these Belgian and French remains will ultimately be referable to cf. *Koalliella* sp. Fragmentary remains from contemporaneous French deposits have also been attributed to a salamandrid [134]; the only preserved vertebra lacks dorsal capping of the neural crest and we regard this material as representing an indeterminate salamandrid.

An exceptional finding is represented by the species *Phosphotriton sigei* from the middle/late Eocene of France [135,136], a mummified salamandrid of uncertain placement. An additional salamandrid, *Megalotriton filholi*, was described from the late Eocene or early Oligocene of France [137,138] (figure 1); it was subsequently reported from the late Eocene of Switzerland [8] and Early Miocene of Spain [139]. According to Estes [8], this taxon shows the closest affinity with *Salamandra sansaniensis* (see below), differentiated by the larger diameter of the transverse processes, cotyle, and condyle of trunk vertebrae in *Megalotriton filholi*. We retain it as a distinct genus, but it could represent the earliest known member of the *Salamandra* lineage.

The published Asian record of Eocene caudates is scarce [12]. It is restricted to the cryptobranchoid *Zaissanurus beliajevae* [140], which is known from specimens from the late Eocene and early Oligocene of Kazakhstan [12] (figure 3*a*).

#### 3.2.6. Oligocene

The most common (extinct) species of Eurasian cryptobranchoid during the late Paleogene–Neogene is the cryptobranchid *Andrias scheuchzeri* [141]. Its stratigraphic range extends from the late Oligocene to the Late Miocene, and it is known from several localities in Germany, Austria and the Czech Republic, as well as Kazakhstan ([140,142,143], see also [12]) (figure 3*a*).

Excluding the two possible Cretaceous occurrences mentioned above, the stratigraphically oldest unequivocal occurrence of a proteid is *Mioproteus gardneri* (figures 1 and 4*a*). This taxon was described from the early Oligocene of Romania by Venczel & Codrea [80] and it appears to be closely related to the extant European genus *Proteus* [59,144].

The salamandrid *Salamandra sansaniensis*, first described by Lartet [145], is one of the most common late Paleogene–Neogene species in Europe (figures 1 and 4*c*). Its stratigraphic range extends from the early Oligocene to the Late Miocene, including several localities in Austria, France, Germany and Spain [8,90,101,103,104,112,114,146–148]. Rage [149] referred a specimen from the middle Eocene of France to *Salamandra sansaniensis*. Although this would not necessitate a large stratigraphic range extension, we are doubtful of its attribution based on an absence of clear *Salamandra* synapomorphies and because of the mainly size-dependent separation between this latter genus and the Eocene taxon *Megalotriton* (see above). *Salamandra sansaniensis* was regarded as the senior synonym of several contemporaneous fossil salamandrid taxa by Estes [8], including *Salamandra broilii*, *Salamandra laticeps*, and the species included in the genera *Heteroclitotriton*, *Dehmiella*, *Voigtiella* and *Palaeosalamandra*, which we broadly follow. However, the synonimization of *Salamandra laticeps*, from the Early Miocene of Germany, with *S. sansaniensis* was questioned by Nussbaum & Brodie [126], who noted similarities between the holotype of the former species (figured by Meyer [123]) and the extant East Asian crocodile newt *Echinotriton*. In particular, the triangular skull of *Salamandra laticeps* is much broader than long, and the size and shape of the ribs are more similar to those typical of the crocodile newt, and, especially, of *Echinotriton*. *Palaeopleurodeles hauffi*, from the late Oligocene of Germany [8,126,150], also shares similarities with *Echinotriton*. As such, we suggest that ‘*Salamandra' laticeps* and *Palaeopleurodeles hauffi* are probable members of this pleurodeline lineage.

The salamandrid *Archaeotriton basalticus*, from the early Oligocene of the Czech Republic and Germany, was described as a *Triturus*-group newt [8,151]. It has very high neural crests in both trunk and caudal vertebrae, with the neural crest twice as high as the remainder of the vertebra [8]. As such, this species might represent an early member of this pleurodeline lineage.

*Brachycormus noachius*, from the late Oligocene of Germany, was initially described by Goldfuss [128], and subsequently by Meyer [123]. A revision of the morphology and systematics of this salamandrid species was presented by Roček [152]. This author also regarded *Tylototriton kosswigi*, from the Oligocene of Germany [124], as a junior synonym of *B. noachius* [152], contrasting with the hypothesis of Nussbaum & Brodie [126] of it pertaining to the extant Asian genus. We agree with the taxonomic proposal of Roček [152], with *Tylototriton* entirely absent from the European fossil record. Two isolated vertebrae from the late Oligocene of Germany have been considered to have affinities with to *Brachycormus* [143]. Böhme [143] noted a clear resemblance between these vertebrae and those of the extant genus *Salamandrina*, but also commented that the absence of a zygosphene/zygantrum complex in the fossil remains excludes attribution to this genus. However, as discussed above, the presence and degree of development of this complex is highly variable in extant *Salamandrina* [153]. Based on the figure published by Böhme [143], we agree that these vertebrae share a striking similarity with those of *Salamandrina*, and could represent its stratigraphically earliest occurrence, prior to its richer Miocene fossil record [154]. These remains are herein attributed to aff. *Salamandrina*, pending further study of *Brachycormus* vertebrae.

Numerous specifically indeterminate remains have been referred to *Triturus* from the late Oligocene to Middle Pleistocene of Austria, the Czech Republic, France, Germany, Hungary, Italy, the Netherlands, Poland, Romania, Russia, Slovakia, the UK and Ukraine [91,101,105,108–112,143,144,155–167] (figure 4*c*). The possible attribution of some of these remains to an extant species should be reassessed, given the high degree of interspecific variation shown by *Triturus* [95,168,169]. Two species referred to *Triturus sensu lato* are based on larval specimens: ‘*Triturus opalinus*' from the late Oligocene of the Czech Republic and *Oligosemia spinosa* from the Late Miocene of Spain [170]. These were instead referred by Estes [8] to *Lissotriton vulgaris* and *Triturus marmoratus*, respectively, but here we regard these as indeterminate Pleurodelinae.

*Triturus rohrsi* was originally described by Herre ([90], figure 6) from the Middle Miocene of the Czech Republic, but the earliest remains are found in the late Oligocene of Germany [143]. After the taxonomic splitting of the *Triturus* genus, this species has been assigned to *Lissotriton rohrsi* [119,143] or *Ommatotriton rohrsi* [171], and we follow the former referral. The species has also been reported from the Early Miocene of Austria [104]. A few remains were referred to an indeterminate species of *Lissotriton*, closely related to *L. rohrsi*, from the Early Miocene of the Czech Republic [110], Middle Miocene of Romania [111] and the Miocene/Pliocene boundary of Greece [119]. We agree with Böhme [143] that vertebrae referred to *T. roehrsi* by Miklas [79] from the Late Miocene of Austria do not belong to this taxon. The dorsal surface of the neural crest of the trunk vertebrae of *Lissotriton rohrsi* is comparable to that of some specimens of the extant species *Lissotriton boscai* (LM, 2022,pers. obs.), although the general proportions of the neural crest, which is especially high and posteriorly narrow, are more similar to those of the vertebrae of the extant genera *Pleurodeles* and *Tylototriton*. The morphology of the neural crest of the trunk vertebrae of *L. rohrsi* is also similar to the Paleocene species *K. genzeli* [89,90]. Although the latter species requires revision, it seems that at least one lineage of newts with robust, capped, and/or ornamented neural crests, morphologically close to the extant crocodile newts from southeast Asia, was widespread in Europe since the Paleocene (figure 1). It is interesting to note that a similarly robust and capped neural crest characterizes the vertebrae of the extant *Salamandrina*, today restricted to Italy, but widespread in Europe during the Miocene [154]. This latter genus could represent a relict of a more widespread lineage of salamanders, as testified by the number of extinct species showing a similar vertebral morphology. However, pending further taxonomic and phylogenetic studies, this hypothesis remains highly speculative, considering that it seems more likely that the robust, capped structure of the neural crest is a plesiomorphic character, secondarily lost in one or more lineages of salamandrids.

Remains from the late Oligocene of Germany were described as *Chioglossa* cf. *meini* by Böhme [143]. This material appears to represent a close relative of the extant Iberian salamandrine species, *Chioglossa lusitanica*. *Chioglossa meini* is otherwise known from the Early and Middle Miocene of the Czech Republic [110] and France, respectively ([101,105], see [172] for an overview of the fossil record of this genus).

#### 3.2.7. Miocene

A third species of *Palaeoproteus*, *P*. *miocenicus*, was recently described by Vasilyan & Yanenko [87] from the Late Miocene of Austria and Ukraine (figure 2*b*). The stratigraphic range of this enigmatic genus is therefore significantly extended by this recent finding (figure 1).

Whereas *Andrias scheuchzeri* (see above) represents the stratigraphically oldest member of Cryptobranchidae present in Europe, it is seemingly not the earliest branching member of the pan-group. *Ukrainurus hypsognathus*, a stem-cryptobranchid, was described from the Middle Miocene of Ukraine by Vasilyan *et al*. [99]. Remains from the Late Miocene of Kazakhstan and two atlantes from the Miocene/Pliocene boundary of Greece also represent indeterminate specimens belonging to Cryptobranchidae [14,119].

The oldest unequivocal occurrences of Hynobiidae are specifically indeterminate remains of the extant genus *Salamandrella* from the late Early Miocene of Russia (figure 2*b*; [173]) and Middle Miocene of Kazakhstan [14]. From the Late Miocene, the fossil record of Hynobiidae becomes relatively rich, including several remains attributable to *Salamandrella* from Kazakhstan, Russia and Ukraine, with the most recent of these dated to the Middle Pleistocene [14,91,174]. The extinct species *Parahynobius kordosi* was described by Venczel [92] from the Late Miocene of Hungary and remains attributed to this genus are present in the Central European record until the Middle Pleistocene [164].

In addition to the aforementioned *Geyeriella wettsteini*, from the Late Miocene of Slovakia [90], the salamandroid species *Mioproteus caucasicus*, first described by Estes & Darevsky [144], has been reported from Middle to Late Miocene deposits in Austria, Hungary, Russia and Ukraine [79,106–108,175]. Several remains referred to indeterminate species of *Mioproteus* have also been documented in the Czech Republic, Moldova, Kazakhstan and Russia, spanning the Early Miocene to the Early Pliocene [14,91,110]. As mentioned above, a distinct proteid genus, *Euronecturus grogu*, more closely related to the North American *Necturus* than to *Mioproteus*, was recently described from the Middle Miocene of Germany [59]. *Orthophyia longa*, from the Middle Miocene of Germany [123], should be considered a nomen dubium, representing an indeterminate proteid [8,59].

The stratigraphically earliest unambiguous occurrence of Plethodontidae is from the Middle Miocene of Slovakia [176] (figures 1*b* and 4*b*). This specimen was attributed to a specifically indeterminate occurrence of the extant genus *Speleomantes*, which is currently found only in Italy and south-eastern France [177].

The only extinct salamandrid species known from China is *Procynops miocenicus* (figure 1). This Early Miocene species was described by Young [178] and shows affinities with the extant Asian genus *Cynops*. The European salamandrid fossil record is much richer, with extinct species and specifically indeterminate occurrences of extant genera reported from the Miocene onwards. Specifically indeterminate occurrences of the extant genus *Mertensiella* have been reported from the Early and Late Miocene of the Czech Republic and Hungary, respectively [110,164]. The extinct species *Mertensiella mera* has been reported from the Early Miocene of the Czech Republic [110], the Early Pliocene of Slovakia (from where it was originally described; [156]) and the Late Pliocene of Poland [155]. This species is regularly found in sympatry with *Chioglossa meini*, and it seems likely that the characters differentiating these two species derive from intraspecific variation [95]. Following Macaluso *et al*. [95], we therefore suggest that *Mertensiella mera* is a junior synonym of *Chioglossa meini*.

Fossils referred to *Salamandra* sp. or the extant species *Salamandra salamandra* (which is smaller and with less evident crests than *S. sansaniensis*) have been described from Late Miocene to Late Pleistocene deposits in Belgium, France, Greece, Hungary, Italy, Poland, Slovakia, Spain and Turkey [119,155,156,164,179–182]. Remains referred to indeterminate species of the genus *Salamandrina* (see above) span the Early Miocene to the Early Pleistocene of Germany, Greece, Hungary, Italy and Spain [119,154,164,183].

Two extinct species of the extant pleurodeline genus *Ichthyosaura* were present in the Miocene of Europe, although remains are scarce and limited to a couple of German localities, represented by *I*. *randeckensis* from the Early/Middle Miocene [184] and *I*. *wintershofi* from the Early Miocene [185]. Marjanović & Witzmann [115] suggested that *I*. *randeckensis* might not be referrable to *Ichthyosaura*, although we retain it in this genus pending further assessment.

*Carpathotriton matraensi* is a newt from the Middle Miocene of Hungary [186,187] (figure 1). The same genus was also identified in the Middle Miocene of Romania [111]. It is characterized by vertebrae with particularly high neural crests, comparable to those of the Oligocene taxon *Archaeotriton basalticus*.

Remains attributed to extant species of *Lissotriton* (or to specifically indeterminate occurrences of this genus) are present in Eurasia from the Early Miocene to the Holocene, documented from localities in Austria, France, Germany, Greece, Hungary, Italy, the Netherlands, Poland, Romania, Russia, Slovakia, Spain and the UK [91,105,106,108,109,111–114,118,119,155–159,164–167,179,183,188–192] (figure 4*c*). Considering the high intra- and interspecific variation seen in extant *Lissotriton*, any specific attribution should be carefully reassessed [95,168,169]. Remains referred to *Triturus sensu lato* from the early Eocene and late Oligocene of France [103,133] might conceivably also belong to this genus.

#### 3.2.8. Pliocene–Pleistocene

*Chelotriton pliocenicus* was described by Bailon [102] from the Late Pliocene of France. The separation from *C. paradoxus* was mainly based on the presence of a zygosphene/zygantrum articulation in *C*. *pliocenicus*. Given considerable variation in this feature in extant species of *Salamandrina* [95,153], it is possible that this is also the case in *Chelotriton*. However, considering the stratigraphic distance between *C. paradoxus* and *C. pliocenicus*, and the absence of a zygosphene/zygantrum complex in specimens of *C. paradoxus* (see [116]), we retain *C. pliocenicus* as a valid species.

Fossil remains referred to the extant salamandrid genus *Pleurodeles* have been reported from the Late Pliocene of Spain [191] and from the Early and Late Pleistocene of Morocco [193]. These areas are within the current geographical range of the extant species *Pleurodeles waltl* [194].

A third species referred to the proteid *Mioproteus*, *Mioproteus wezei*, has been reported from the Pliocene of Poland, France, and Russia [160,195,196] and Early Pleistocene of Moldova [197] (figure 4*a*). *Mioproteus wezei* was erroneously reported to be present in the Pleistocene of Poland in figure 5 of Macaluso *et al*. [59], but this locality (Weze II) is actually Late Pliocene in age (MN 16 according to Młynarski *et al*. [195] and Stefaniak [198]). The proteid *Proteus bavaricus* and an indeterminate sirenid, from the Pleistocene of Germany, were described by Brunner [199] based on parasphenoids. These remains are doubtfully attributed and probably do not belong to caudates, with a teleostean attribution previously suggested [8,59].

A second extinct species of the hynobiid *Parahynobius* (*P*. *betfianus*) was described by Venczel [92] from the Middle Pleistocene of Romania (figure 1). Averianov & Tjutkova [200] assigned material from the Early Pleistocene of Kazakhstan to the extant Central Asian salamander genus, as *Ranodon* cf. *sibiricus*. Specifically indeterminate remains of *Speleomantes* have been reported from the Early and Late Pleistocene of Italy [161,177,201]. Remains comparable to and possibly referable to the extant species *Ichthyosaura alpestris* have been reported from the Early Pleistocene of Italy [201] and from the Middle Pleistocene of Russia [91]. The extant salamandrid *Ommatotriton* was reported from the Middle Pleistocene of Israel [202]. A Late Pleistocene*–*Holocene Japanese locality yielded several isolated remains referred to the two extant species *Cynops ensicauda* and *Echinotriton andersoni* [203], representing the first fossils unequivocally attributed to these genera.

## 4. Discussion

### 4.1. Spatio-temporal sampling of the Palearctic caudate fossil record

Based on the number of occurrences in each time bin (figure 5), it is evident that our knowledge of the fossil record of Palearctic caudates is biased by the fragmentary and spatio-temporally heterogeneous nature of the fossil record (e.g. [204]). In particular, not all time bins are equally well-represented, with both the stratigraphically earliest and the most recent intervals the least sampled for caudates. The first and last intervals of the evolutionary history of a taxon are characterized by sampled diversity that is often lower than ‘true' biological diversity (e.g. [205]), and thus this might account for part of the pattern we observe. Therefore, the restriction of the fossil record of early caudates to the Eurasian species *Triassurus sixtelae* prior to the Middle Jurassic does not necessarily reflect the true diversity of Palearctic caudates at this time. There is a relatively rich terrestrial tetrapod fossil record from the Late Triassic of Europe and Early Jurassic of Asia (e.g. [206,207]), but this does not typically represent the mesic environments which caudates (and other amphibians) occupied. Although it is possible that caudate diversity remained genuinely depauperate in the Palearctic during the group's early evolutionary history, which is consistent with an effective slow initial radiation of the three modern orders of amphibians, as suggested by molecular data [208], their absence might instead reflect a sampling bias, with the Middle Jurassic ‘radiation' merely reflecting increased sampling of mesic environments.

The highest taxa to localities ratios in the Mesozoic occur in the Middle Jurassic and (to a lesser extent) the Early Cretaceous. Whereas this Mesozoic record is dominated by Asian occurrences, the Cenozoic record of caudates is predominantly from Europe (with the exception of the Early Pleistocene). This pattern might be explained by a combination of geographical and anthropogenic biases. Continental outcrops are relatively uncommon in Europe for much of the Jurassic and Cretaceous (e.g. [209]), and mainly limited to its western part (figure 2). By contrast, not only is Asia characterized by a more extensive continental record for this interval (e.g. [210]), it also preserves several Lagerstätten. These deposits have received a large amount of attention because of the exquisitely preserved dinosaur and mammal fossils they have yielded [211–214], but they have also produced numerous, exceptionally preserved specimens of caudates (e.g. [37,40,42]). No comparable Lagerstätten are known from the Jurassic or Cretaceous of Europe (with the exception of Las Hoyas in Spain; [215]). By contrast, the Cenozoic continental record of Europe is rich, but there is also an extensive Asian record. The description of fossil caudates from Asia has recently escalated, with 81% of papers describing caudate fossils from Asia published in the last two decades. We propose that the relatively depauperate fossil record of caudates in the Cenozoic of Asia likely reflects a collector or describer bias. For example, several cryptobranchoid fossil remains from the Cenozoic of Asia have been mentioned in the literature, but have either not been formally described and/or figured, or have yet to be referred to a particular species (e.g. [12] for Cryptobranchidae; [216] for Hynobiidae). This is supported by molecular analyses that suggest a Cenozoic diversification of Hynobiidae, coeval with the initial uplift of the Tibetan Plateau, 50 Ma [217]. Zhang *et al*. [217] suggested that a high-mountain, stream-type lifestyle that characterizes most extant hynobiid species is ancestral for the group, with the low-land, pond-type adaptation of some living taxa (e.g. *Salamandrella*) evolving later. A prominent taphonomic bias therefore might affect the fossil record of hynobiids, as suitable depositional environments are extremely rare in mountain habitats [218]. It is possible that the appearance of crown hynobiids in the fossil record in the Middle Miocene (figure 3*b*) coincided with the evolution of pond-like adaptations in the group and thus increased their preservation potential.

The general pattern during the early Paleogene is of low observed diversity. This might be a genuine reflection of a long recovery period in the aftermath of the K/Pg mass extinction, with caudates severely affected by this event [219,220]. Nevertheless, both the Paleocene and Eocene are poorly sampled (figure 5), and so this low diversity could be partly artefactual. Most of the remains from these two time-intervals are referred to extinct taxa (e.g. *Chelotriton* and *Koalliella*; figure 1), including more inclusive taxonomic groups with no living representatives (e.g. Batrachosauroididae; figure 1). The increasing diversity observed in the Oligocene coincides with the first appearance of most of the extant genera of salamandrids. This interval is characterized by an increase in speciation rates and a decline in extinction rates in caudates [220], and it is therefore likely that the Oligocene represents a genuine interval of heightened diversification in this clade relative to that of the early Paleogene.

During the Neogene and Quaternary, Palearctic caudates known from articulated skeletal remains are proportionally rare, whereas they are much more common in the Mesozoic and Paleogene (figure 6). The lack of articulated skeletons constitutes a problem for determining the taxonomic identity of specimens, as well as for understanding the phylogenetic relationships of extinct taxa known from limited skeletal remains. For example, most phylogenetic analyses that incorporate fossil taxa only include species known from essentially complete skeletons (e.g. [30,115,187]). Furthermore, taphonomic biases influence the preservation of disarticulated remains, with the largest and most robust elements more often represented than other parts of the skeleton in palaeontological collections [95,153]. As such, limb and girdle elements are often found as fragmentary bones that do not bear diagnostic features and are thus consequently identified as indeterminate caudate or vertebrate remains, whereas vertebrae are more commonly identified, described and attributed to a specific taxon (figure 7). However, osteological studies of extant Palearctic species are limited as far as the vertebrae are concerned, with a greater emphasis on limbs (nearly exclusively as proxies for age determination through skeletochronology) and the skull (figure 7). The lower jaw is also not extensively studied in extant species, preventing a more comprehensive understanding of the relationship between fossil and present-day caudate occurrences. Projects such as the *Global Herpetological Osteology* initiative [15], which aims to evaluate how much is known about the osteology of extant species of reptiles and amphibians, might be essential for understanding how and where palaeontological and osteological research should be oriented.

The low sampling of recent time bins reflects a common trend in palaeoherpetology. In many groups (e.g. lizards and turtles; [221,222]), including caudates, the highest observed diversity of Eurasian taxa occurs in the Miocene, with a substantial reduction in the Pliocene (figure 5). It is likely that a true biological diversity pattern is recognizable herein: molecular analyses indicate that the last major radiations in caudates occurred in the Late Miocene [220]. Furthermore, there was a substantial turnover in Palearctic terrestrial faunas in the latest Miocene to earliest Pliocene, with the extinction of numerous species, including mammals (e.g. [223,224]), squamates [222], turtles [221] and crocodylians (e.g. [225–227]), in addition to caudates. However, based on the number of localities from the Early, Middle and Late Miocene (figure 5), it is evident that this epoch is far better sampled than previous and subsequent ones. Thus, at least part of this trend could pertain to sampling bias, with continental Pliocene outcrops less well-sampled than their Miocene counterparts. This interpretation is supported by the ratio of the number of taxa to the number of localities per time bin, with the Miocene ratio equivalent to that of the Pliocene (figure 5). Furthermore, Villa & Delfino [222] suggested that potentially rich Pliocene localities are indeed present, but that their palaeoherpetofaunas often remain unstudied or unexcavated, with a collector bias towards other taxa (e.g. mammals) and stratigraphically earlier time intervals yielding extinct taxa. Once ‘corrected' for the number of localities (figure 5), diversity in the Early and Middle Pleistocene is not substantially different from that of the Pliocene, whereas it is particularly low in the Middle/Late Pleistocene, suggesting that biodiversity might genuinely have been low at this time. Present-day caudate diversity is depauperate when compared with the Miocene. Given that sampling-corrected diversity indicates little change in late Neogene to early Quaternary Palearctic caudate diversity, it is probable that low present-day diversity was primarily shaped by extinction in the Late Pleistocene. This was probably the result of increased severity of climatic oscillations during this interval, both in terms of temperature and frequency of the shifts [228].

### 4.2. Implications for the biogeographical history of Palearctic caudates

Our review and evaluation of the fossil record of Palearctic caudates documents the extinction of many species and several groups, including the demise of numerous taxa known from the Plio-Pleistocene. Furthermore, it reveals that caudate clades were more broadly distributed in the Palearctic in the past, with the present-day distribution of some extant taxa contracted relative to that of their recent past (figures 2–4).

Stem urodeles and karaurids are represented in western Asia and the UK in the Middle Jurassic, demonstrating that non-urodele caudates were already widespread by this time interval (figure 2*a*). This is consistent with the hypothesis of a diversification and distribution across Laurasia during the Early Jurassic [229,230]. The Asian origin of Cryptobranchoidea, already suggested by several authors (e.g. [229]), is supported by the presence of the putative stem-member *Nuominerpeton aquilonaris* from the Early Cretaceous of China [30] (figure 3*a*). The fossil record of Cryptobranchidae, as currently understood, would suggest a progressive colonization of western areas, first appearing in the Cretaceous of western Asia, but only reaching Europe by the Oligocene (figure 3*a*), where this family is now extirpated. By contrast, the fossil record of Hynobiidae is poorer and the biogeographic pattern less evident, as unequivocal representatives of the crown group do not appear anywhere in the fossil record until the Miocene [173]. However, the first hynobiid remains are from the Middle Miocene of western Asia, suggesting also in this case an east-to-west colonization of the western Palearctic (figure 3*b*; see above for the taphonomic biases that might affect the fossil record of Hynobiidae).

Stem salamandroids are rare in the Palearctic fossil record. The only taxon supported as a stem salamandroid by phylogenetic analyses [30] is *Valdotriton* from the Early Cretaceous Spain, and a possible stem salamandroid position is herein suggested for the contemporaneous UK genus *Apricosiren*, as well as *Kiyatriton* from the Middle Jurassic–Early Cretaceous of Russia, although these taxa are only known from disarticulated elements. As such, the scarce record prevents any possible consideration on the Palearctic biogeographic history of the clade Salamandroidea.

The extant salamandroid clade Ambystomatidae is today restricted to North America, and there is no current support for its presence in the Palearctic fossil record (contra [8]). Although the highest known diversity of the extinct salamandroid group Batrachosauroididae comes from North America, the stratigraphically oldest putative remains are from the Early Cretaceous of Europe [47], predating the earliest North American remains (Late Cretaceous; [231]). As such, it remains possible that Batrachosauroididae originated in Europe and subsequently dispersed into North America.

Fossil remains unequivocally attributed to Plethodontidae are extremely rare, limited to the Middle Miocene of Slovakia [176] as well as the Pleistocene of northern Italy and southern France [177,201] (figure 4*b*). All the European records of plethodontids are characterized by the presence of amphicoelous vertebrae. This vertebral morphology is rare in extant members of this family and in North American members it is only present in a few genera [53]. However, the two extant Eurasian genera, *Speleomantes* (closely related to the North American genus *Hydromantes*) and *Karsenia*, share this vertebral morphology. These two extant taxa have a disjunct distribution, with *Speleomantes* restricted to northern and central Italy (Sardinia island included) and southern France, and *Karsenia* present in South Korea [232]. Based on their recovery as sister taxa, Vieites *et al*. [232] suggested that the most parsimonious biogeographic scenario for the Eurasian plethodontids is that they originated in northeastern Asia, from where *Speleomantes* dispersed to western Eurasia. However, more recent analyses do not support a close relationship between *Karsenia* and *Hydromantes*/*Speleomantes* [233] and the scenario proposed by Vieites *et al*. [232] is therefore not supported. The fossil record of plethodontids is too poor to test other biogeographic scenarios (for a comprehensive discussion on the biogeography of this group predating the discovery of *Karsenia*, see [177]).

Fossil remains of Proteidae are widely distributed in Europe and western Asia from the Oligocene onwards, and they are also found in Miocene outcrops in Asia (figure 4*a*). The oldest specimen attributed to this family is the putative proteid vertebra from the Early Cretaceous of Europe ([54]; see above for the taxonomic assignment) that predates the oldest North American occurrence (*Paranecturus garbanii* from the Late Cretaceous; [234]), suggesting a possible European origin for this clade. The low number of localities yielding unambiguous proteid remains in North America (only three localities; [58,96,234]) might also support this hypothesis. However, given the strong bias affecting the fossil record of this group (composed entirely of isolated remains and mostly vertebrae) and the consequent lack of a robust understanding of its phylogenetic relationships, any biogeographic scenario is tentative. Proteids are entirely absent from Asia today and their European distribution is restricted to the extant species *Proteus anguinus* in the Balkan Peninsula [235]. This follows the extinction of the Eurasian genus *Mioproteus* by the Pleistocene [91,160,197], possibly caused by the climatic oscillations that severely affected many European reptiles and amphibians ([236] and references therein).

Fossil Salamandridae show mainly European occurrences (figure 4*c*), with a rich and widespread fossil record from the Paleocene [10]. Such a high diversity seems to confirm the hypothesis of a European origin for the clade, as suggested by several previous works (e.g. [229,237]). The first taxa that occur in central and eastern Asia are *Procynops* (Early Miocene of China), closely related to the extant Chinese genus *Cynops*, and *Chelotriton* (present in central Asia in the Early and Middle Miocene), which shows close similarities to the extant crocodile newts *Tylototriton* and *Echinotriton* from southeast Asia [94]. This implies that the expansion to the east of Salamandridae happened not later than the Miocene, with subsequent extirpation from central Asia. This extirpation, that led to the present-day disjunct distribution of Eurasian salamandrids, might have been caused by increased aridity and the resultant expansion of the steppe-desert biome in the Late Miocene as a consequence of Tibetan uplift ([238]; see also [239]).

## 5. Conclusion

The fossil record of Palearctic caudates is not evenly distributed through time or space. Taxa known from articulated skeletal remains or imprints on slabs are proportionally rare, with isolated remains common, especially vertebrae. This constitutes a problem for taxonomic identification, as well as for reconstructing the evolutionary interrelationships of caudates, especially as phylogenetic datasets primarily focus on species with relatively completely known skeletons.

The stratigraphically oldest known caudate worldwide is from the Middle/Late Triassic of central Asia and, following a substantial stratigraphic gap, stem-Urodela and Karauridae are present in both western Asia and the UK in the Middle Jurassic. High observed diversity in this latter time bin, together with that of the latest Early Cretaceous, is driven primarily by Asian Lagerstätten. The apparent radiation of caudates in the Middle Jurassic might be an artefact of a lack of sampling of suitable mesic depositional environments earlier in the Mesozoic, although it remains possible that the early evolution of amphibians is characterized by low diversification rates.

A possible member of stem-Cryptobranchoidea has been reported from the Early Cretaceous of eastern Asia, prompting suggestions of an Asiatic origin of this clade. Only a few species of Cryptobranchidae were present in Europe, first appearing here in the Oligocene, and were subsequently extirpated on this continent, as well as in western and central Asia. Salamandrids appear to have originated in Europe, expanding into Asia by the Miocene. Their disjunct present-day distribution reflects local extirpations, with a likely role for climatic and habitat changes linked to the uplift of the Tibetan Plateau. Within the Palearctic, the extinct family Batrachosauroididae is only reported from Europe, and the same is true at the moment for the fossil occurrences of the extant family Plethodontidae. Fossil remains of proteids are present in Europe and central Asia from the Oligocene, showing a strong range contraction by the Pleistocene, to their present-day restriction to the Balkan Peninsula. The Miocene provides a rich caudate record, with an apparent decline in diversity at the beginning of the Pliocene. However, this decline appears to be mainly an artefact of uneven sampling. Instead, low present-day caudate diversity in the Palearctic was primarily shaped by climatically driven extinction and range contraction in the Late Pleistocene.

## Data Availability

The data are provided in electronic supplementary material [240].
